# Treatment and Prognosis for Pancreatic Duct Disruption Associated With Pancreatic Cancer: A Case Series of 15 Patients

**DOI:** 10.7759/cureus.67482

**Published:** 2024-08-22

**Authors:** Yuki Oka, Takeshi Tanaka, Takashi Kobayashi, Atsuhiro Masuda, Arata Sakai, Masahiro Tsujimae, Masanori Gonda, Hirochika Toyama, Takumi Fukumoto, Yuzo Kodama

**Affiliations:** 1 Division of Gastroenterology, Department of Internal Medicine, Kobe University Graduate School of Medicine, Kobe, JPN; 2 Division of Hepato-Biliary-Pancreatic Surgery, Department of Surgery, Kobe University Graduate School of Medicine, Kobe, JPN

**Keywords:** endoscopic retrograde pancreatography, pancreatic pseudocyst, endoscopic treatment, pancreatic cancer, pancreatic ductal disruption

## Abstract

Background

Pancreatic duct (PD) disruption can occasionally be attributed to pancreatic cancer. Therapeutic interventions for PD disruption due to pancreatic cancer and their influence on pancreatic cancer prognosis remain unclear. This study investigated the therapeutic modalities and prognostic implications of PD disruption in pancreatic cancer.

Methods

This retrospective study included 15 patients with PD disruption concomitant with pancreatic cancer between April 2011 and March 2023. As an endoscopic intervention for PD disruption, endoscopic pancreatic stenting (EPS) or endoscopic ultrasonography-guided pancreatic fluid collection drainage (EUS-PFD) was performed. Technical success was defined as stent placement and clinical success was defined as an improvement in PD disruption.

Results

Of the 15 cases of PD disruption, two involved only pancreatic juice leakage without symptoms, four involved pancreatic pseudocyst (PPC) without infection, and nine involved PPC with infection. Four patients underwent EPS, nine underwent EUS-PFD, and two underwent lumen-apposing metal stent placement. All patients achieved both technical and clinical success without complications. The clinical stage of pancreatic cancer ranged from carcinoma in situ to the metastatic phase. For the treatment of pancreatic cancer, five patients underwent surgical resection, and eight underwent chemotherapy. There was no obvious recurrence of peritoneal sowing. The median overall survival from the diagnosis of pancreatic cancer in the resected and non-resected cases was 74 and 9.6 months, respectively.

Conclusion

Endoscopic intervention was effective in all cases of PD disruption due to pancreatic cancer. Furthermore, even in cases of pancreatic cancer after PD disruption, survival rates were similar to those in cases without PD disruption and were achieved through surgical resection or chemotherapy.

## Introduction

Pancreatic ductal disruption arises from injuries to the pancreatic duct (PD), such as acute pancreatitis, chronic pancreatitis, surgical interventions, trauma, and pancreatic tumors [1-5]. PD disruption leads to the leakage of pancreatic juice, resulting in various complications, including pancreatic ascites, pancreatic pleural effusion, fistula, and abscess formation. Notably, the formation of pancreatic pseudocyst (PPC) is the most common consequence of pancreatic juice leakage [1,2,6].

For the treatment of PD disruption or PPC, endoscopic interventions are invaluable. Endoscopic retrograde pancreatography (ERP) is useful in identifying PD injuries and treating PD disruptions through endoscopic pancreatic stenting (EPS) [6]. In cases of symptomatic PPC, endoscopic ultrasonography-guided pancreatic fluid collection drainage (EUS-PFD) is a highly efficacious approach [7].

There have been several reports on PD disruption and PPC formation caused by pancreatic cancer [8-16]. When pancreatic cancer coexists, it is necessary to address not only PD disruption but also to devise a therapeutic strategy for the pancreatic cancer itself. However, an efficacious treatment for pancreatic cancer-associated PD disruption remains unclear. Furthermore, the prognosis of patients with pancreatic cancer concomitant with PD disruption, and whether pancreatic juice leakage poses a risk for peritoneal dissemination, have not been clarified. In this case series, we aimed to investigate the effectiveness of interventions for PD disruption associated with pancreatic cancer and the treatment modalities and prognosis of pancreatic cancer. This article was previously posted to the Research Square preprint server on April 22, 2024.

## Materials and methods

Patients database

A retrospective review of the endoscopic database at Kobe University Graduate School of Medicine in Kobe, Hyogo, Japan, identified 60 patients who underwent endoscopic intervention for PD disruption between April 2011 and March 2023. Cases attributed to inflammatory etiologies such as acute or chronic pancreatitis, postoperative complications, or trauma were excluded from the study. Finally, 15 patients underwent endoscopic treatment specifically for PD disruption associated with pancreatic cancer. We collected clinical information, including sex, age, body mass index (BMI), lifestyle history, medical history, symptoms, blood test results, imaging findings, endoscopic treatment details, and tumor treatment history. The study protocol was approved by the Kobe University School of Medicine Ethics Committee (no. B232030). All authors had access to the study data and reviewed and approved the final manuscript.

Endoscopic treatment

Computed tomography (CT) was performed before the initiation of endoscopic treatment for PD disruption. EPS was performed in cases where cyst formation was absent or inaccessible via the gastrointestinal tract. Using a TJF Q290V or 260V endoscope (Olympus Optical, Tokyo, Japan), a five- or seven-French pancreatic PS was inserted to bridge the PD disruption. If it was not possible to place a PS across the site of PD disruption, the PS was positioned in proximity to the disrupted site.

EUS-PFD was performed when the cysts displayed distinct encapsulation and were accessible via the gastrointestinal tract. Using a GF-UCT260 linear echoendoscope (Olympus Optical, Tokyo, Japan), several seven French, 4 to 7 cm double pigtail PS were inserted into patients with pseudocysts, with the addition of a nasocystic drainage catheter in select cases. In some infected PPC cases, lumen-apposing metal stents (LAMS) were placed, and repeated DEN was performed as required. After verification of PPC reduction using CT, the LAMS was removed.

The timing of PS removal or replacement was considered within three months of ERP or EUS-PFD after confirming symptom improvement and cyst shrinkage; however, the final decision was left to the discretion of the attending physician. Each procedure involves multiple physicians, including trainers and trainees.

Definitions of pancreatic duct disruption and outcomes

PD disruption was defined as the leakage of contrast agent outside the PD during ERP, the presence of pancreatic enzyme-rich fluid drainage from EUS-PFD, or the loss of continuity in the PD, accompanied by fluid accumulation in its vicinity as visualized by imaging methods. Technical success in endoscopic interventions was defined as the successful placement of a plastic stent (PS) or LAMS using a TJF endoscope or GF a UCT260 linear echoendoscope. According to a previous study [17], clinical success in the improvement of PD disruption was defined as the resolution of symptoms or successful resolution of pancreatic fluid collection with asymptomatic cases. Infection of the PPC was defined as the presence of fever, elevated C-reactive protein (CRP) level, or the detection of bacteria within the PPC.

## Results

Patient characteristics

The characteristics of the 15 patients are shown in Table 1.

**Table 1:** Patient characteristics BMI: body mass index; CA19-9: carbohydrate antigen 19-9; CEA: carcinoembryonic antigen; CRP: C-reactive protein; PDAC: pancreatic ductal adenocarcinoma; TP: total protein

Characteristic	All patients (n=15)
Age, yr, median (range)	64 (45-71)
BMI, g/m^2^, median (range)	18.6 (13.6-26.7)
Sex, male, n (%)	10 (66.6%)
Alcohol consumption <50 g/day, n (%)	5 (33.3%)
History of smoking, presence, n (%)	8 (53.3%)
Family history of PDAC, presence, n (%)	2 (13.3%)
Diabetes mellitus, presence, n (%)	1 (6.6%)
Chronic pancreatitis, presence, n (%)	0 (0.0%)
Symptoms	13 (86.7%)
Abdominal pain, presence, n (%)	12 (80.0%)
Fever, presence, n (%)	4 (26.7%)
CRP, mg/dl, median (range)	5.9 (0.07-18.5)
TP, g/dl, median (range)	6.6 (6.2-7.4)
CEA ng/ml >5.0, n (%)	5 (33.3%)
CA19-9 U/ml >37, n (%)	12 (80.0%)

The median age was 64 (range: 45-71), and among the 15 patients, 10 were male. The median CRP level was 5.9 mg/dL (range: 0.07-18.5), and the median total protein was 6.6 g/dl (range: 6.2-7.4). Thirteen of the 15 patients exhibited symptomatic presentations, with 12 reporting abdominal pain and four presenting with fever. Carcinoembryonic antigen and carbohydrate antigen 19-9 levels exceeded the normal threshold in five (33.3%) and 12 (80.0%) cases, respectively.

Features of pancreatic duct disruption and endoscopic treatment

The details of PD disruption and endoscopic treatment are presented in Table 2.

**Table 2:** Pancreatic duct disruption and treatment EPS: endoscopic pancreatic stenting; LAMS: lumen-apposing metal stent; PD: pancreatic duct; PFD: pancreatic fluid collection drainage; PPC: pancreatic pseudocyst; PS: plastic stent

Pancreatic duct disruption and treatment	All patients (n=15)
Disruption site, n (%)	
Pancreatic head	1 (6.6%)
Pancreatic body	7 (47.7%)
Pancreatic tail	7 (47.7%)
Tumor and disruption site, n (%)	
Proximal site to tumor	6 (40.0%)
Caudal site from tumor	9 (60.0%)
Phenotype of PD disruption	
Pancreatic juice leakage only	2 (13.3%)
PPC with infection	9 (60.0%)
PPC without infection	4 (26.7%)
Long diameter of cyst, mm, median (range)	75 (16-174)
Endoscopic treatment, n (%)	
EPS	4 (26.7%)
PFD with PS	9 (60.0%)
PFD with LAMS	2 (13.3%)
Technical success	15 (100%)
Clinical success	15 (100%)
Complications by endoscopic treatment	0 (0.0%)
Median time to clinical success, days, median (range)	12.5 (2-46)
PS removal, n (%)	10 (66.7%)
Median time to PS removal, days, median (range)	23 (6-293)

The site of rupture in PD was identified as the head, body, and tail of the pancreas in one, seven, and seven cases, respectively. Disruption was observed proximal to the tumor in six cases, while nine cases exhibited caudal disruption from the tumor. There were two cases with only pancreatic juice leakage, Four PPC without infection, and nine cases of PPC with infection. The median of long cyst diameter was 75 mm (range: 16-174). Additionally, one patient presented with a concurrent mediastinal pancreatic fistula. No cases of disconnected PD syndrome were observed.

Technical and clinical success was achieved without complications in all 15 patients. EPS was performed in four cases. Of the four cases treated with EPS, bridging of the ruptured site could not be performed in two cases. However, placing the EPS close to the disruption site yielded a sufficient therapeutic effect. Nine patients with EUS-PFD underwent PS. Among them, an additional nasocystic drainage catheter was placed in eight cases. For the two patients with PPC, a LAMS was inserted. In one case employing LAMS, an attempt at DEN was hindered owing to the lack of space to insert the endoscope, and the PS was placed after the LAMS was removed. The median time to clinical success after endoscopic treatment was 12.5 days (range: 2-46 days). No recurrence of PD disruption was observed after endoscopic treatment. PS was removed in 10 cases, and the median time to PS removal was 23 days (range: 6-293 days).

Diagnosis of pancreatic cancer

Diagnoses of pancreatic cancer are presented in Table 3.

**Table 3:** Diagnosis and treatments for pancreatic cancer EUS-FNA: endoscopic ultrasonography-fine needle aspiration; IPMC: intraductal papillary mucinous carcinoma; PD: pancreatic duct; PDAC: pancreatic ductal adenocarcinoma; UICC: Union for International Cancer Control

Diagnosis and treatments for pancreatic cancer	All patients (n=15)
Diagnosis of pancreatic cancer, n (%)	
Before PD disruption	6 (40.0%)
At the same time of PD disruption	8 (53.3%)
After PD disruption	1 (6.7%)
Histological diagnosis, n (%)	
Pancreatic juice cytology	5 (33.3%)
EUS-FNA	9 (60.0%)
None	1 (6.7%)
Diagnosis of pancreatic cancer	
PDAC	13 (86.7%)
IPMC	2 (13.3%)
Clinical stage (UICC 8th), n (%)	
0	2 (13.3%)
I	2 (13.3%)
II	1 (6.7%)
III	4 (26.7%)
IV	6 (40.0%)
Treatment for pancreatic cancer, n (%)	
Surgical resection	5 (33.3%)
Chemotherapy	8 (53.3%)
Best supportive care	2 (13.3%)

Six cases of PD disruption developed during chemotherapy, eight cases manifested pancreatic cancer diagnosis within one month of PD disruption onset, and in one case, pancreatic cancer was diagnosed 200 days after the PD disruption.

All six cases receiving chemotherapy had already been diagnosed with pancreatic ductal adenocarcinoma (PDAC) by EUS-fine needle aspiration (FNA). Among the eight cases diagnosed with pancreatic cancer within one month of PD disruption, five received a conclusive diagnosis through ERP, while three cases underwent diagnostic confirmation via EUS-FNA. In one case, diagnosis was difficult through ERP; however, a conclusive diagnosis of pancreatic cancer was successfully attained through surgical resection. One required approximately 200 days from the occurrence of PD disruption to the diagnosis of pancreatic cancer because of treatment for PPC.

**Figure 1:**
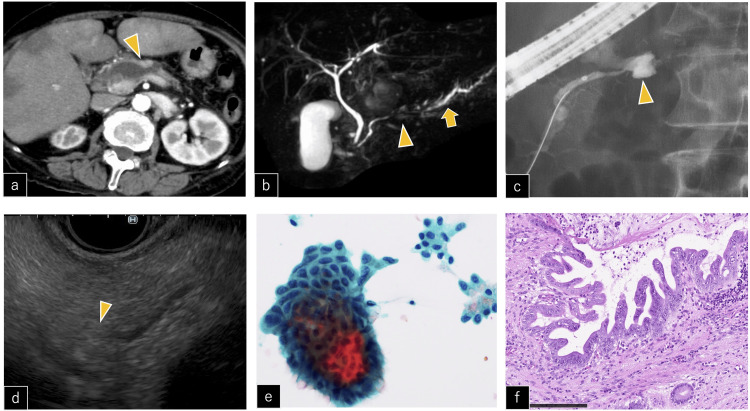
CIS with PD disruption a) CE-CT image showing loss of continuity of the pancreatic duct (arrowhead); b) MRCP resonance cholangiopancreatography showing MPD stricture (arrowhead) and dilation of the caudal MPD (arrow); c) ERP showing leakage of the contrast agent outside the MPD (arrowhead); d) EUS image showing no mass in the MPD stricture (arrowhead); e) pancreatic juice cytology revealed atypical cell clusters with enlarged, irregularly sized, and unevenly distributed nuclei; adenocarcinoma was suspected; f) the surgical specimen showed enlarged, irregular nuclei and disturbed cell polarity and was diagnosed as CIS (H&E, x40). Scale bars, 200 μm. CE-CT: contrast-enhanced computed tomography; CIS: carcinoma in situ; ERP: endoscopic retrograde pancreatography; EUS: endoscopic ultrasonography; H&E: hematoxylin and eosin; MRCP: magnetic resonance cholangiopancreatography; MPD: main pancreatic duct; PD: pancreatic duct

Among the cohort of 15 cases of pancreatic cancer, two cases were intraductal papillary mucinous carcinoma (IPMC), while the remaining cases were PDAC. At the time of pancreatic cancer diagnosis, the staging was as follows: two cases at stage 0, two cases at stage I, one case at stage II, four cases at stage III, and six cases at stage IV. There was a carcinoma in situ (Figure 1) and one case of microscopic pancreatic cancer smaller than 10 mm that could not be detected on CT.

Treatment for pancreatic cancer and overall survival

The treatment options for pancreatic cancer are presented in Table 3. According to the National Comprehensive Cancer Network guidelines [18], after the diagnosis and treatment of PD disruption, five cases underwent surgical resection, eight underwent chemotherapy, and two received best supportive care (BSC). The median time from the first endoscopic treatment to surgery was 48.5 days (range: 19-1520).

Among the five cases who underwent surgical resection, one was resected after treatment for PPC, and none experienced complications graded as 2 or higher within the initial 30-day postoperative period according to the Clavien-Dindo classification [18]. In all five cases, peritoneal lavage cytology results were negative. Moreover, no clear dissemination nodules or significant ascites accumulation indicative of peritoneal dissemination recurrence were observed after surgery. However, among the five surgically resected cases, two exhibited pancreatic recurrence (528 and 1287 days after surgery), one had liver metastasis (563 days after surgery), and one had lung metastasis (358 days after surgery).

The median time from the first endoscopic treatment to chemotherapy in the eight chemotherapy-treated patients was 28 days (range: 16-166 days). The median duration of continued chemotherapy was 214 days (range: 71-886). Two patients opted for the best supportive care after treatment for PD disruption without the desire for chemotherapy.

The median overall survival from the diagnosis of pancreatic cancer in the resected and non-resected cases was 74 and 9.6 months, respectively.

## Discussion

PD disruptions associated with pancreatic cancer requiring endoscopic intervention are rare, with only a few cases reported [8-16]. In this case study, we demonstrated that pancreatic cancer can be effectively managed with appropriate treatment of PD disruption, resulting in a prognosis consistent with previous reports [19]. Among the five surgically resected cases, intraoperative peritoneal lavage cytology results were negative. Additionally, no clear dissemination nodules or significant ascites accumulation indicative of peritoneal dissemination recurrence were observed post-surgery.

PD rupture due to pancreatic cancer is thought to result from increased intraductal pressure [12]. One reason for the low incidence of PD rupture in pancreatic cancer is the gradual progression of ductal obstruction. As the tumor size increases, caudal pancreatic atrophy ensues, leading to a reduction in pancreatic juice production [16]. In these 15 cases, there was no evidence of pancreatic atrophy, suggesting that pancreatic fluid production may not have been reduced. Additionally, two of the 15 cases involved IPMC. IPMC is characterized by the formation of a mass within the main PD, and it was believed that the tumors physically disrupted the PD.

Tyberg et al. reported that PD disruption could be safely and effectively treated using various endoscopic procedures [7]. In this study, both the technical and clinical success of endoscopic treatment were achieved in all cases. Shrode et al. reported that the disruption of the main PD should be managed using bridging stenting [19]. Although bridging stenting was prioritized in two of the five cases treated with EPS, bridging stent placement could not be achieved. Therefore, the stent was placed proximal to the PD rupture, and the rupture could be effectively treated. Decreasing the duodenal pressure gradient of the PD by placing the EPS near the disruption site may be sufficient therapy [6]. For the majority of PPC, EUS drainage has been reported to allow for safer access and a decrease in complications [7].

In this study, the patients could receive appropriate surgery or chemotherapy after endoscopic treatment for PD disruption. Adverse effects of surgery are feared due to intra-abdominal adhesions associated with PD disruption [20]. However, all patients who underwent resection could leave the hospital without postoperative complications. Chemotherapy was continued in eight patients. Although there was a case in which the pancreatic fistula spread to the mediastinum and thoracic cavity, resulting in postoperative pulmonary metastasis, there were no cases of obvious peritoneal dissemination recurrence. The median overall survival in this study was comparable to that previously reported for pancreatic cancer in Japan [19]. It has been reported that clinically relevant postoperative pancreatic fistulae after resection for pancreatic cancer are significantly associated with worse overall and disease-free survival [21]. However, future case studies are needed to determine whether preoperative pancreatic pleural effusion or pancreatic ascites affect recurrence.

Limitations

The primary limitations of our study are that it was a single-center retrospective study and that the number of cases was small. Another limitation of this study is the absence of a comparison cohort. Consequently, we were unable to directly compare the outcomes of endoscopic treatment for PD disruption in cases without pancreatic cancer.

## Conclusions

Endoscopic treatment was effective and safe in all cases of pancreatic cancer-associated PD disruption. Even in the presence of cyst infection along with PD disruption, pancreatic cancer can be treated after appropriate management with endoscopic therapy. Furthermore, the prognosis of pancreatic cancer was comparable to that of pancreatic cancer without PD disruption. Due to the rarity of PD disruption associated with pancreatic cancer, careful follow-up is essential in managing PD failure of unknown etiology. This should include cytological diagnosis and imaging studies, given the potential for coexisting pancreatic cancer.
